# Hypercalcaemia after treatment with denosumab in children: bisphosphonates as an option for therapy and prevention?

**DOI:** 10.1007/s12519-020-00378-w

**Published:** 2020-08-10

**Authors:** Carmen Sydlik, Hans Roland Dürr, Susanne Bechtold-Dalla Pozza, Claudia Weißenbacher, Julia Roeb, Heinrich Schmidt

**Affiliations:** 1 Department of Pediatric Endocrinology, Dr. v. Haunersches Children’s Hospital, Ludwig-Maximilian-University of Munich, Lindwurmstr. 4, 80337 Munich, Germany; 2 Department of Orthopaedics, Physical Medicine and Rehabilitation, University Hospital, Ludwig-Maximilian-University of Munich, Munich, Germany

**Keywords:** Aneurysmatic bone cyst, Bisphosphonates, Calcium homeostasis, Denosumab, Giant cell tumor of the bone

## Abstract

**Background:**

Pharmacologic options for treatment of osteolytic diseases especially in children are limited. Although not licensed for use, denosumab, a fully humanized antibody to RANKL, is used in children with good effects. Among others, one possible indication are giant cell tumors and aneurysmatic bone cysts. However, there are reports of severe hypercalcemia during weeks to months after termination of denosumab, that are rarely seen in adults.

**Methods:**

We collected data of four patients, aged 6–17 years, who experienced severe hypercalcemia after completion of treatment with denosumab for unresectable giant cell tumors of bone or aneurysmal bone cysts and methods of their treatment. The detailed case information were described.

**Results:**

One patient was treated with long-term, high-dose steroid therapy, leading to typical Cushing’s syndrome. Another patient was restarted on denosumab repeatedly due to relapses of hypercalcemia after every stop. Finally, in two patients, hypercalcemia ceased definitely after treatment with bisphosphonates. However, several applications were necessary to stabilize calcium levels.

**Conclusions:**

There is a considerable risk of hypercalcemia as an adverse effect after denosumab treatment in children. Therapeutic and, preferably, preventive strategies are needed. Bisphosphonates seem to be an option for both, but effective proceedings still remain to be established.

## Introduction

Treatment of osteolytic diseases is known to be difficult as pharmacological possibilities are limited, particularly in children. For several years now, denosumab, a fully humanized antibody to RANKL, is available for treatment of postmenopausal osteoporosis and skeletal related events caused by bone metastases of certain solid tumors in adults [1]. It is an effective and quite frequently used therapeutic option, also as an alternative to bisphosphonates because of its faster treatment effect. By binding to RANKL (“receptor activator of NFkappaB-ligand”) with very high affinity, denosumab strongly blocks its interaction with RANK and thereby inhibits the formation of osteoclasts as well as their differentiation and activation. As a consequence, bone resorption is inhibited, resulting in an increase in bone mass [1–4]. In contrast, bisphosphonates block osteoclast activity, but not their development [1, 4–6]. Because of their strong adherence to hydroxylapatit, they remain in the bones for many years [1, 4–6], raising concerns about long-term consequences especially when used in children [7]. For denosumab, effects seem to be more temporary [3], but severe hypercalcemia may develop once treatment is stopped owing to a rapid loss of newly acquired bone caused by rebound formation and to activity of osteoclasts [3, 8–10].

In children, there currently is no approved indication for denosumab; however, it is used as an individual strategy or in clinical trials for several indications, especially certain forms of osteogenesis imperfecta [11–13]. Denosumab is also used in the treatment of multiostotic fibrous dysplasia, juvenile Paget, giant cell tumors, aneurysmatic bone cysts and others [4, 9, 14–19]. Moreover, two NCT trial studies for the use of denosumab in children with giant cell tumor of bone are on their way [20] or have recently completed data acquisition [21].

Giant cell tumors and aneurysmatic bone cysts are similar, though not identical, expansive bone lesions that may occur in any bone of the body, being most prevalent in femur, tibia, radius, and pelvis. Although benign, these tumors and cysts can be locally very aggressive in their osteolytic activity and may cause significant bone destruction with sequelae of pain, weakness, deformity and pathological fractures [8]. Surgery is the treatment of choice for giant cell tumors; whereas, non-surgical options are used in the treatment of aneurysmal bone cysts [10, 22, 23], because epimetaphyseal (complete) resection is often impossible [8, 10, 17]. Histologically, giant cell tumors consist of sheets of neoplastic ovoid mononuclear cells interspersed with osteoclast-like giant cells expressing RANKL. Recruitment of osteoclast-like giant cells is thought to be related to the expression of RANKL on some mononuclear (stromal) cells, which are the actual tumor cells. Therefore, the giant cells themselves are responsible for the aggressive osteolytic activity of the tumor [8, 10]. By taking these pathophysiological conditions into account, denosumab was thought to be a good therapeutic opportunity for irresectable giant cell tumors. Several trials confirmed its efficacy [8, 10, 19, 23], and denosumab has now been approved for treatment of giant cell tumors in adults in the USA [10]. Although these tumors are most frequent in young adulthood (3rd and 4th decade) [4, 17], they rarely occur in children and adolescents. There are several case reports of successful treatment with denosumab as well as ongoing trials in this age group [4, 9, 14, 16, 17, 19–21]. Independently of the indication for its use, the risk of hypocalcaemia immediately after start of treatment with denosumab caused by increased calcium sequestration in bone is well-known, and calcium and vitamin D supplementation for its prevention is generally recommended now [15]. In contrast, along with the above-mentioned notions of reactively increased osteoclast activity after cessation of denosumab, most of the reports on children mention hypercalcaemic episodes [4, 9, 14, 15, 18, 19], which are often difficult to treat. These sequelae have not yet been systematically addressed in a clinical study. Nevertheless, with the increased off-label-use in children and with the severe symptoms of hypercalcaemia in most reports, we think it is necessary to be concerned about this topic. By presenting the cases of four children (Table 1), we would like to discuss possible options for therapy and, more importantly, the prevention of post-denosumab hypercalcaemia.

**Table 1:** Patient characteristics

Patient	age at diagnosis (y)	Indication	Duration of denosumab treatment (mon)	Doses of denosumab 60 mg	Operative intervention	Interval: end of denosumab and hypercalcemia	Treatment of hypercalcemia	Time to relapse (d)
1	12	ABC	17	17	None	1 mon	BP	14
2	12	GCT	14	14	Tumor resection	2 mon	Long-term steroids	None
3	6	ABC	9	12	Embolisation	3 mon	Recurrently denosumab	Not exactly known
4	13	GCT	14 and 7	17 and 10	Partial resection and stabilisation of the spine (twice)	2–3 mon; in spite of “precautional” BP	BP	14 and 10

*ABC* aneurysmatic bone cyst, *GCT* giant cell tumor, *BP* bisphosphonates

## Methods

Four children, aged 6–17 years, presented to our children’s hospital for severe hypercalcemia after treatment with denosumab for unresectable giant cell tumors of bone and for aneurysmal bone cysts. Therapy with denosumab had been started between 09/2011 and 12/2014. In all patients, the same treatment regimen for denosumab with a dose of 60 mg on days 1, 8, 15, 28, and then once a month had initially been used. Data on their medical course and on methods used for treatment of hypercalcemia were collected for each patient and were described as case reports.

## Results

### Patient 1

**Fig. 1:**
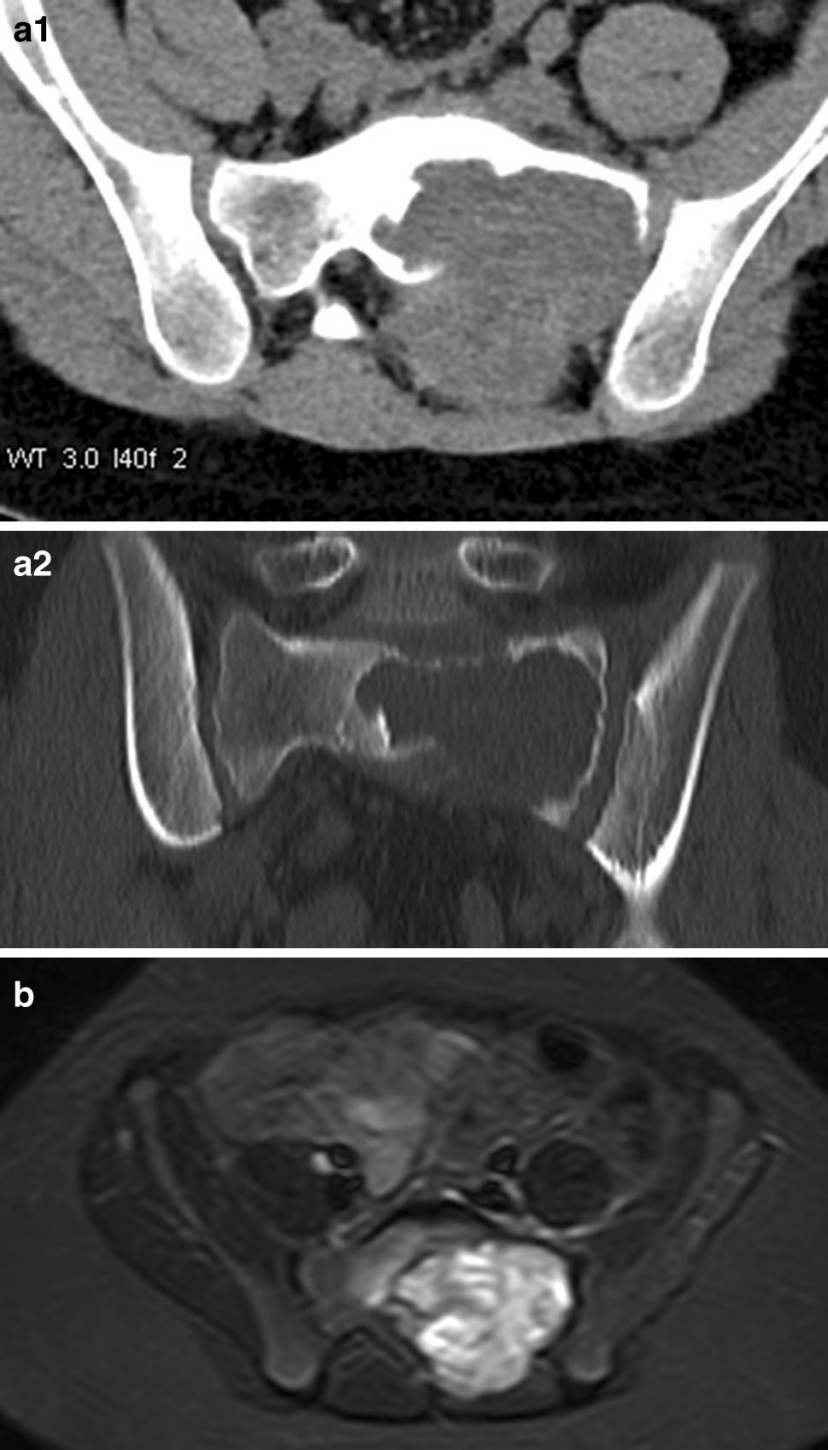
Diagnostic imaging of patient 1. **a1**, **a2** Initial computer tomography scan. **b** magnetic resonance tomography after 11 months of denosumab treatment

**Fig. 2:**
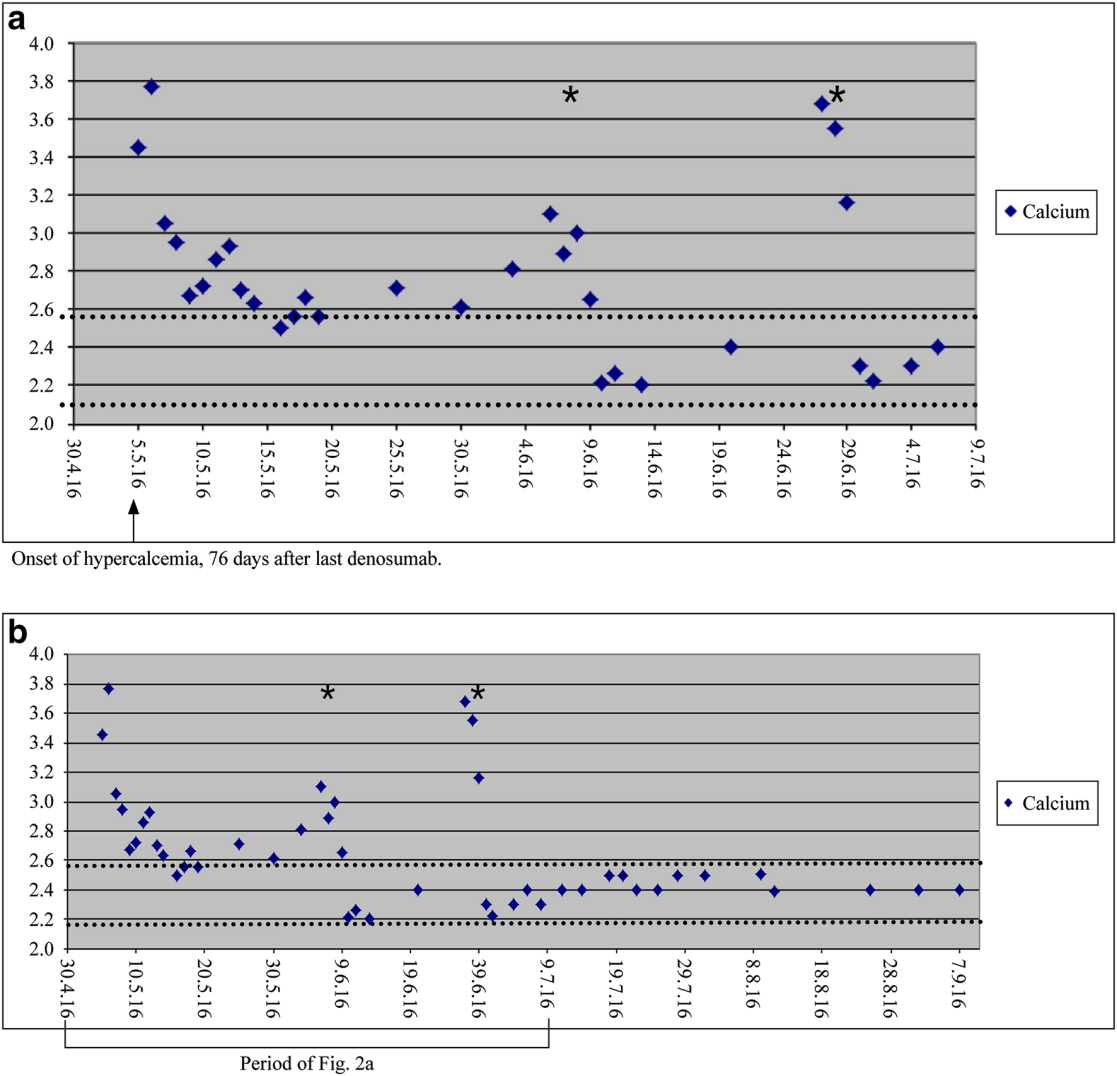
Calcium levels of patient 1. **a** During acute phase of hypercalcemia. **b** During hypercalcemia and 3 month follow-up. Dotted lines: normal range of calcium; asterixes: bisphosphonate doses

In October 2014, an 11-year-old boy was diagnosed with a solid variant of aneurysmatic bone cyst in the left os sacrum (Fig. 1a1, a2). The tumor caused intermittent pain in the left hip and leg. Surgical treatment was difficult owing to the localisation and extension of the tumor; therefore, off-label-use of denosumab was started in November 2014 at a dose of 60 mg on days 1, 8, 15, 28, and then once a month. When MRI and CT controls showed ossification of the lesion (Fig. 1b), denosumab treatment was gradually tapered by extending the interval between the doses from 4 to 8 weeks. Denosumab treatment was stopped after 16 months of treatment. Two and a half months (76 days) after the last injection of denosumab, the boy presented to a peripheral children’s hospital with dizziness, nausea, vomiting, and abdominal and generalized pain. Laboratory findings showed severe hypercalcaemia (3.95 mmol/L). The boy was treated with hydration, furosemide, and corticosteroids. Symptoms and laboratory values improved only slightly and worsened again after 1 week; therefore, the boy was referred to our university children’s clinic. Initial blood tests showed a calcium level of 3.77 mmol/L. Given that other causes could be excluded, the hypercalcaemia was interpreted as a consequence of rebound increase of osteoclast activity following the denosumab therapy. To stop osteoclast hyperactivity, we decided to administer bisphosphonates. After one infusion of neridronate 2 mg/kg, calcium level returned to lower-normal values (2.21 mmol/L) within 1 day and remained low for the next several days. During the following days all other medications (hyperhydration, furosemide, prednisolone) could be stopped. However, 2 weeks later, the boy was readmitted to our hospital with the previous symptoms of hypercalcaemia. Though tight ambulant calcium controls had still shown normal values 2 days before, calcium had risen to 3.68 mmol/L again. A second dose of neridronate 2 mg/kg was given with the same effect as before (calcium: 2.33 mmol/L 2 days later) (Fig. 2a). Afterwards, tight calcium controls always showed normal results for the following 6 months and also a year later, and parents reported no recurrence of symptoms (Fig. 2a, b). The aneurysmatic bone cyst remained stable and partially calcified.

### Patient 2

In December 2014 a giant cell tumor in lumbosacral spine (L5/S1) was found in a girl aged 11 years. She was treated with denosumab starting in December 2014 with the same dose regime as in patient 1 for 12 months. When the tumor had diminished in size sufficiently, it could be curreted and denosumab treatment was tapered with extending intervals from 1–2 months, ending December 2015. Approximately 3 months after the last dose of denosumab, the girl started to vomit and experienced nausea and abdominal pain. When she presented to a primary children’s clinic, she had hypercalcaemia (maximal level: 3.93 mmol/L) and prerenal renal failure. As with patient 1, rebound bone resorption after stopping denosumab therapy was found to be the only explanation. After treatment with hydration and furosemide failed to be effective, a high dose of prednisolone was started and was successful to normalize hypercalcaemia and to restore renal function. Aiming to prevent recurrent hypercalcemia, prednisolone treatment was continued at a lowered dose. One half year later, the dose of prednisolone was still as high as 0.25 mg/kg/d and had caused relevant Cushing syndrome with weight gain, striae, weakness, and slight arterial hypertension. The girl was sent to our endocrinology department with a request for safely reducing the cortisone treatment. Dose was tapered very slowly combined with tight calcium controls. Dose reduction was tolerated well, and no hypercalcemia occurred. When cortisone therapy could be stopped (December 2016), Trab5b, a marker of osteoclast activity, showed a normal level of 19 U/L (normal range: 9–22 U/L). Three months later, calcium level was still normal. Nevertheless, symptoms of Cushing’s syndrome were still present. Also, there was no recurrence of the giant cell tumor.

### Patient 3

In July 2011, a 6-year-old child of Russian origin presented with pain and reduced sensitivity in his left thigh. A core needle biopsy was used to diagnose an aneurismal bone cyst with typical osteoclast-like giant cells and intense vascularisation. Curettage and filling with bone chips was considered dangerous owing to the risk of bleeding; therefore, treatment with denosumab was chosen as the first-line treatment. Within 2 months after the start of denosumab in September 2011 (60 mg every 4 weeks with two additional doses on days 8 and 15), the boy was free of pain and a control-CT-scan showed increasing bone density at the margins of the lesion. Then, an additional embolisation was performed. Treatment with denosumab was continued for 1 year till September 2012. The boy’s family returned home to Russia. Three months after denosumab was stopped, the boy was admitted to a hospital in Russia because of dizziness, vomiting, lack of appetite, and tiredness. Laboratory investigations showed hypercalcemia of 4.14 mmol/L and metabolic alkalosis. The Russian colleagues restarted denosumab applications with 30 mg every third month for 10 months until September 2013. However, once this treatment was stopped, the boy developed another phase of hypercalcemia and a further one, after the latter had again resolved by a third course of denosumab. In January 2016, the boy was under stable condition with no further hypercalcemic events, no progression of the aneurysmal bone cyst, and no complaints.

### Patient 4

In the fourth patient, a giant cell tumor localised in Th2, complicated by pulmonary metastasis, was identified in November 2012 at the age of 13 years. Owing to osteodestruction of Th2 and to compression of the spinal channel, the patient was treated with denosumab (same dose regime with 60 mg on days 1, 8, 15, 28, and then once a month) from December 2012 until January 2014. Dorsal decompression and spondylodesis of thoracic vertebra 1–3 could then be performed, as well as diagnostic partial lung resection on the right side and further resection of the giant cell tumor. After eighteen months (in August 2016), recurrent problems occurred which requiring the correction of spondylodesis of cervical vertebra 7 to thoracic vertebra 4. For further stabilisation, the boy was treated with denosumab again for 7 months (September 2016–April 2017). With the experience of patient 1’s hypercalcemia in mind, patient 4 was given a dose of bisphosphonates (ibandronate) in the orthopaedic department immediately after stopping denosumab with the hope of preventing hypercalcemia in April 2017.

**Fig. 3:**
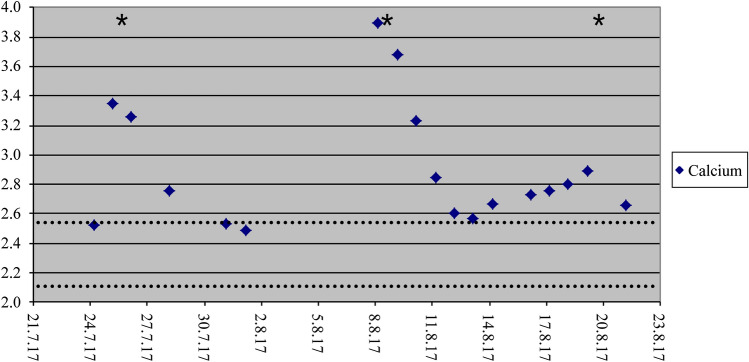
Calcium levels of patient 4. Dotted lines: normal range of calcium; asterixes: bisphosphonate doses

However, in July 2017, (i.e., approximately 3 months after the final denosumab injection) the boy, aged 17 years, presented to a peripheral clinic with symptoms including dizziness, weakness, and vomiting for several weeks. Laboratory results showed hypercalcemia > 4 mmol/L, which had developed in spite of the precautional bisphosphonate treatment, and did not respond to conventional therapy with furosemide and prednisolone. Moreover, the boy showed mild renal failure, nephrocalcinosis stage II, and elevated blood pressure. As a consequence, he was transferred to the intensive care unit of our university children’s hospital, where bisphosphonates (neridronat 2 mg/kg) were applied again. Two days later, calcium returned to normal, and the boy wasdischarged. Unfortunately, the boy had to be readmitted 14 days after the infusion of neridronate owing to recurrent dizziness and vomiting. Calcium was elevated again with 3.90 mmol/L. After another dose of neridronat (2 mg/kg), calcium levels dropped, but only to 2.85 mmol/L. Keeping the high cumulative amount of bisphosphonates in mind, we gave neridronate again 10 days later, but reduced in dose (1 mg/kg). Finally the boy could be discharged then with at least high-normal calcium levels (2.59 mmol/L; Fig. 3). Calcium levels remained in the normal range for the following 2 months, but 3 months later computer tomography showed significant progression of the giant cell tumor as well as the pulmonary metastases, so denosumab was restarted (60 mg every 3 months). Meanwhile, with stabilisation of the lesions, the treatment is tapered again, but is still ongoing.

## Discussion

Few reports exist on hypercalcemia after treatment with denosumab, as seen in the four patients presented here. Initially, Gossai et al. [14] as well as Setsu et al. [4] described hypercalcemia in children treated with denosumab for giant cell tumors. Uday et al. [18] added case reports of two more adolescents and a 40-year-old man. Grasemann et al. [13] found this side effect in a child with juvenile Paget, and Boyce et al. [9] in a boy with severe fibrous dysplasia both treated with denosumab. Recently, Kurucu et al. [19] and Dürr et al. [24] reported reactive hypercalcemia in several denosumab-treated children and adolescents with aneurysmatic bone cysts, the phenomenon clearly must be related to the treatment.

To explain these observations, the following pathopyhsiologic hypothesis was proposed. According to the mechanostat model [25, 26], the body has a certain setpoint for the adequate quantity of bone for its muscle mass and physiological needs. The newly acquired bone during denosumab treatment is not limited to local lesions, such as aneurysmatic cysts or fibrous deformities. Because of the lack of osteoclast activity, bone mass cannot be adapted to the physiologic needs and is judged as being excessive. With cessation of denosumab treatment, the inhibitory effect on osteoclasts stops, resulting in a reactive exaggerated activity of these cells [4, 14]. Kurucu et al. [19] could prove these theoretic approaches to be true by showing not only suppressed parathormone but also high deoxypyridinoline levels indicating a rebound increase in osteoclastic activity. The resulting surplus of bone resorption allows a high amount of calcium ions to be set free, leading to hypercalcaemia. In adults, a transient rise of bone turnover markers after cessation of denosumab treatment has been reported several times [8–10, 26, 27] and can be regarded as a proof of this theory. In older patients treated mainly (but of course, not exclusively) for osteoporosis, these observations lead to concerns that the effect of gaining bone mass by denosumab might soon be lost afterwards [8–10]; however, there are only few case reports of symptomatic hypercalcemia in adults that stopped treatment [5, 18]. On one hand, this might be due to the lack of reports on follow-up, which also do not exist for many case presentations in children; on the other hand, a higher incidence in children might be explained by a generally more active turnover and by acquisition of bone in growing children and adolescents [9].

This phenomenon is additionally supported by the observation of increases in calcium and bone resorption markers in children with osteogenesis imperfecta at the end of the interval before their next dose of denosumab [28]. Their lower rate of hypercalcemia might be due to their generalized low bone mass and at least in part, that they were pretreated with bisphosphonates. In giant cell tumors or in aneurysmatic bone cysts instead, there is only a local deficiency of bone. Moreover, in the studies of patients treated for osteogenesis imperfecta, doses of denosumab were much lower (1 mg/kg/dose) [11, 28] than in the reports on giant cell tumors and aneurysmatic bone cysts in children. In them, mainly the standard dosage for adults adapted from a phase 2 clinical trial in adults [29] were used. As a result, the four patients described in our case report received approximately 2-3 mg/kg/dose (exact data cannot be given as initial weight was unfortunately not available). Nevertheless, in the already mentioned children with juvenile Paget [15] and fibrous dysplasia [9], symptomatic hypercalcemia occurred in spite of lower doses (1.0 mg/kg, then increased to 1.75 mg/kg and 0.5 mg/kg). This implies that while dose regimes might play a role for the individual risk of hypercalcemia, other, thus far unknown, factors must coexist. The impossibility of predicting individual risk for hypercalcemia after denosumab treatment and the extent of hypercalcemia is shown impressively by patient 4, in whom no hypercalcemia was seen after the first, but after the second treatment. A subclinical hypercalcemia after the first use, of course, cannot be excluded, even more as nephrocalcinosis stage II was already present at onset of the adverse event after the second phase.

Hypercalcemia must certainly be considered a serious adverse event, as all of the children reported with hypercalcemia after cessation of denosumab showed quite severe symptoms, such as fatigue, nausea, vomiting, constipation, abdominal pain, weight loss, polyuria, dehydration, renal failure, and sinusbradycardia (1 report), which often developed within a few days [4, 9, 14, 15]. Fortunately, thus far they did not, but could, in part, become life-threatening.

Therefore, to underscore demands already raised by Uday et al. [18], it seems to be necessary to develop safety concepts for weight-adjusted dosing, frequency and duration of therapeutic use of denosumab in children. Additionally, monitoring calcium levels at the end of treatment should be mandatory.

Given the current state of knowledge, neither the risk nor the exact timing of onset of hypercalcemia can be anticipated for a single individual. There are no data on real frequency of these sequelae. So far, there are only two case series reports of children with and without hypercalcemia after treatment [19, 24]. An incidence of 22, respectively, 20% could be calculated, but, of course, the sample size is too small to generalize these data. The interval between the last dose of denosumab and hypercalcemia was between 2.5 and 3 months in all of the patients reported here, regardless if treatment had been tapered to 2-monthly intervals (patient 2 and 3) or continued monthly till the end (patient 3 and 4). Others [4, 14, 15, 18, 19] reported an interval of 7 weeks to 5 months, although it has to be added that the child in which hypercalcemia occurred after 7 weeks was still on substitution with calcium because of initial hypocalcemia after denosumab injections. The mean half-life of denosumab is of about 29 days (range 25–35 days) [30], but it may be prolonged in individuals with accumulated doses [18]. As a result, duration of denosumab treatment might also be relevant for the timing of onset of hypercalcemia. Our patients were treated between 7 and 16 months. The treatments of other patients lasted between 14 and 24 months [4, 14, 18, 19]. From these few reports, we cannot conclude with certainty that a correlation between duration of therapy and time to onset of hypercalcemia exists. An additional influencing factor, among others, might also be the individual vitamin D level [18].

Most important, however, is the urgent need to evolve effective strategies to manage these hypercalcaemic episodes. In the patients reported here and by others, “conventional” methods like hydration combined with diuretics and short-time corticosteroids showed no or only transient effects. To our knowledge, there is no evidence that high-dose steroids for a prolonged interval, as in patient 2, would really be effective to surpass the interval till the reactive bone resorption would probably have declined. Short- and long-time sequelae of Cushing syndrome should be kept in mind. Restarting denosumab also appears not to be the solution (e.g., patient 3). Instead, bisphosphonates (single dose or repeated), which were finally used in almost all reported patients [4, 5, 9, 14, 15, 18], led to normalization of calcium.

Additionally, prevention of hypercalcemia in denosumab-treated children should be of higher interest. Thus far, there are no reports in the literature concerning this topic. In our opinion, bisphosphonates might be helpful. Although denosumab has actually been used in several cases in children when bisphosphonates failed to achieve the expected effect, the combination with bisphosphonates is worth considering: one of the modes of action of bisphosphonates is their long-lasting adherence to bone matrix and by this preventing bone resorption. Therefore, bisphosphonates also could reduce the activity of the reactively formed new osteoclasts after cessation of denosumab therapy and consequently could avoid severe hypercalcemia and its symptoms. This was the hypothesis we used—to our knowledge for the first time—to give a dose of bisphosphonates to prevent hypercalcemia in patient 4. Unfortunately, our attempt was not successful, but in our opinion, it is nevertheless worth considering further. Maybe the time point and/or frequency or even dosage of bisphosphonates require adjustment. Urgency of further research on this topic is impressively shown by patient 4.

For future patients on denosumab, pretreatment with bisphosphonates might be a possible option for prevention of hypercalcemia. But as some of the children in whom hypercalcaemia was observed had formerly been treated with bisphosphonates, also the time interval to the start and end of denosumab seems to matter. As well pretreatment with bisphosphonates combined with a dose soon after stopping denosumab and/or another one about 1 month later could be a strategy worth trying.

In conclusion, although denosumab is not yet licensed in children and adolescents, more and more studies revealed it is efficacious not only in giant cell tumors and aneurysmatic bone cysts but also in several other osteolytic diseases. However, and different from adults, severe hypercalcaemia after the end of the treatment seems to be rather frequent, due to the generally higher bone turnover in growing children. In our opinion, it is crucial to think about strategies of treatment and even more, prevention. Bisphosphonates could be an option for both. To find the right timing and dose regimens for preventive use, more studies are needed.
